# Mitogenomic divergence between three pairs of putative geminate fishes from Panama

**DOI:** 10.1080/23802359.2017.1413288

**Published:** 2017-12-12

**Authors:** Fernando Alda, Alexandria J. Adams, Prosanta Chakrabarty, W. Owen McMillan

**Affiliations:** a Museum of Natural Science, Department of Biological Sciences, Louisiana State University, Baton Rouge, LA, USA;; b Smithsonian Tropical Research Institute, Panama, Republic of Panama

**Keywords:** Gobiiformes, Isthmus of Panama, mitogenome, peripheral fishes

## Abstract

We sequenced the complete mitochondrial genomes of three pairs of congeneric peripheral fishes distributed on either side of the Isthmus of Panama in order to test their status as geminate species pairs. Our phylogenetic analysis did not support a sister relationship between *Gobiomorus dormitor* and *G. maculatus* and therefore they cannot be considered geminates. The average genetic distance of protein-coding genes between *Sicydium altum* and *S. salvini* was more than two times larger than between Atlantic and Pacific *Awaous banana*, suggesting different timings for their divergence across the Isthmus of Panama.

## Introduction

The formation of the Isthmus of Panama represents one of the most dramatic geological events in Earth’s history with major climatic and biological implications. Despite its global importance, the timing of the uplift of the Isthmus of Panama remains an open question that has resulted in a thrilling scientific discussion (Montes et al. 2012; Bacon et al. 2015a, 2015b; Lessios 2015; O’Dea et al. 2016; Jaramillo et al. 2017), in which the most recent studies challenge the long-held idea of a Pliocene connection (ca. 3.5 Million years ago) of North and South America (Coates and Obando 1996). Thus, recent works propose an earlier age (i.e. Miocene-Oligocene) for the formation of the Isthmus, as well as a more complex geological history than previously believed (Montes et al. 2012).

The formation of the Isthmus created a landbridge that allowed species to cross between South and North America, and, at the same time, created a barrier to gene flow between marine organisms in the Pacific and Caribbean. Provided this barrier, species may have diverged on either side of the Isthmus into descendant sister taxa called ‘geminate species’ (Jordan 1908). Molecular evidence indicates that all geminate species were not simultaneously separated by the emerging Isthmus (Knowlton and Weigt 1998; Marko 2002; Lessios 2008; Miura et al. 2010; O’Dea et al. 2016), and suggests that ecological differences, such as habitat preference, may influence the timing of separation. For example, mangrove dwelling invertebrates exhibit fewer genetic differences than species associated with deeper habitats (Knowlton and Weigt 1998; Miura et al. 2010). Similarly, peripheral fishes (i.e. groups with a recent marine origin that have invaded coastal streams; Myers 1949) of the genus *Dormitator* show remarkably young divergence times (Galván-Quesada et al. 2016). These results support the idea that near-shore taxa are ideal candidates for molecular clock calibrations, because their limited genetic divergences might reflect the latter stages of transisthmian marine connection (Knowlton and Weigt 1998; Galván-Quesada et al. 2016).

In this study, we provide a comparison of genetic distances across complete mitochondrial genomes of three pairs of peripheral fish species, or lineages, with distributions on either side of the Isthmus of Panama. We infer their evolutionary relationships to test the hypothesis that they are geminate species.

## Materials and methods

We collected specimens from six species or lineages of gobiiform fishes distributed in Atlantic and Pacific watersheds of Panama: *Gobiomorus dormitor* (STRI-15207, Río Chagres, Río Chagres, Panama Province, Panama), *Gobiomorus maculatus* (STRI-7077, Río Pavo, Río Pavo, Veraguas Province, Panama), *Sicydium altum* (STRI-4981, Río Bongie, Río Changuinola, Bocas del Toro Province, Panama), *S. salvini* (STRI-660, Río Chiriquí Viejo, Río Chiriquí Viejo, Chiriquí Province, Panama; STRI-3170, Río Lajas, Río Santa María, Herrera Province, Panama), *Awaous banana* (STRI-4986, Río Bongie, Río Changuinola, Bocas del Toro Province, Panama), and *A. banana* (STRI-11209, Río Mamoní, Río Bayano, Panama Province, Panama). We excised the gill arches and preserved them in a 20% dimethyl sulphoxide (DMSO), 0.5M EDTA, pH 8 solution at 4 °C until DNA extraction. All specimens were initially fixed in formalin, later transferred to 70% ethanol, and deposited and vouchered in the Neotropical Fish Collection (NFC-STRI) at the Smithsonian Tropical Research Institute in Panama (STRI).

We recovered complete mitochondrial DNA sequences as a by-product of hybrid target capture of ultraconserved elements (UCEs) (Faircloth et al. 2012). Briefly, we extracted DNA using a QIAGEN DNeasy kit (Qiagen, Valencia, CA) and randomly sheared 400–1000 ng of DNA by sonication to a target size of 400–600 base pairs (bp). We constructed genomic libraries for each of our samples using the Kapa Hyper Prep Kit v.3.15 (Kapa Biosystems, Wilmington, MA), and enriched them for 500 UCE loci using the Actinops-UCE-0.5Kv1 probe set (Faircloth et al. 2013) following protocols for the MYcroarray MYBaits kit v.3.0 (MYcroarray, Ann Arbor, MI). Then we sequenced these libraries in one lane of PE150 Illumina MiSeq (Illumina, San Diego, CA).

We trimmed raw reads for adapter contamination and low quality bases in illumiprocessor (Faircloth 2013), and mapped them to reference mitogenomes using Bowtie 2 (Langmead and Salzberg 2012) as implemented in Geneious 10.1.3 (www.geneious.com, Kearse et al. 2012). Also using Geneious 10.1.3, we created and annotated consensus sequences.

We aligned our mitogenomes with representative mitogenomes from 22 species belonging to five out of the seven families of Gobiiformes (sensu Agorreta et al. 2013), including three newly sequenced mitogenomes of Neotropical eleotrids (Alda et al. 2017), and *Kurtus gulliveri* (Kurtiformes) that was used as an outgroup. We extracted and individually re-aligned all 13 protein-coding genes for subsequent phylogenetic analysis. We partitioned the data by gene and by codon, and estimated the best partition scheme under the GTR + GAMMA substitution model using PartitionFinder (Lanfear et al. 2012). We used these partitions in a Maximum Likelihood analysis in RAxML (Stamatakis 2014), in which we ran 40 searches to find the best tree, and performed 500 bootstrap replicates to assess nodal support.

To compare mitochondrial divergences across pairs of putative geminate taxa, we calculated uncorrected p-distances between congeneric taxon pairs for each mitochondrial gene, and calculated their standard errors using 500 bootstrap replicates in MEGA7 (Kumar et al. 2016).

## Results and discussion

We obtained complete mitochondrial genome sequences with a minimum of 10× coverage for three species pairs, or lineages, of peripheral gobiiform fishes: *G. dormitor* (MF927493) and *G. maculatus* (MF927494); *S. altum* (MF927496) and *S. salvini* (MF927497, MF927498); and *A. banana* from Atlantic (MF927488) and Pacific (MF927489) watersheds.

The size of the mitochondrial genomes ranged from 16,474 bp in *G. dormitor* to 16,536 bp *in G. maculatus*. All species contained 22 tRNA genes, two rRNA genes, 13 protein coding regions, as well as control region arranged in the same order. The most common start codon ATG was found in all genes except in COXI that had the start codon GTG, and in ND6 that had TAC as the start codon. The stop codon TAA terminated the genes ND2, ND4L and ATP8 in all species, whereas genes ND1 and ND5 exhibited either TAA or TAG as stop codons. Incomplete stop codons were found in some or all species for genes ATP6, COXII, COXIII, CYTB, ND3 and ND4 formed of only T or TA, which are likely completed as TAA via post-transcriptional polyadenylation.

**Figure 1.**
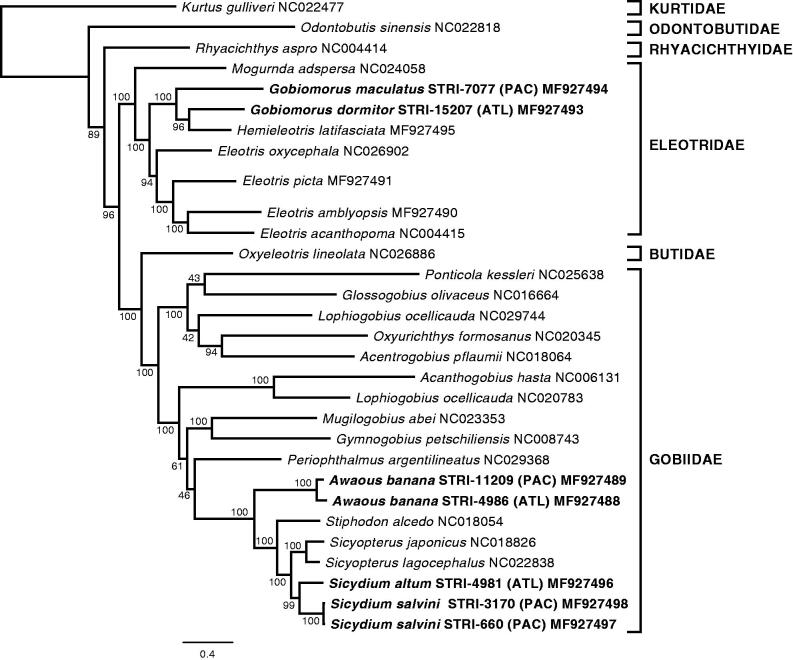
Maximum-likelihood (RAxML) phylogeny using 13 protein-coding mitochondrial genes from a selection of species of Gobiiformes (GenBank accession numbers indicated), and *Kurtus gulliveri*, which was used as the outgroup. Numbers next to nodes are support values obtained after 500 bootstrap replicates. Sequences from specimens obtained in this study are highlighted in bold.

Our phylogenetic analysis was congruent in the relationships among families and genera with recent studies using both mitochondrial and nuclear loci (Agorreta et al. 2013; Adrian-Kalchhauser et al. 2017). The species or lineage pairs of *Sicydium* and *Awaous* showed sister relationships and can be considered as geminate species. However, *G. dormitor* and *G. maculatus* were not recovered as sister species, and consequently the hypothesis that they are geminate species was rejected (Figure 1). This result is in contrast with the results of Thacker and Hardman (2005) and Thacker (2017), who analyzed the same data set in a phylogeny of the Gobiodei and in a comparative morphological analysis of geminate species, using Maximum Parsimony and Bayesian Inference, respectively. In both cases they recovered a sister relationship for the two species of *Gobiomorus*. Conversely, further reanalyses of the data from Thacker and Hardman (2005) have also evidenced the non-monophyly of *Gobiomorus*, and similarly to our study, have recovered a sister relationship between *G. dormitor* and *H. latifasciata* (Thacker 2009; Agorreta and Rüber 2012). Furthermore, and in agreement with the latter, a recent barcoding study with a broader geographical and taxonomical sampling within the Eleotridae, pointed to the Eastern Pacific species *G. polylepis* as the sister of *G. dormitor*, and *H. latifasciata* as sister to the two of them (Guimarães-Costa et al. 2017). According to this, *G. dormitor* and *G. polylepis* would constitute the geminate species pair in the genus *Gobiomorus*.

**Figure 2.**
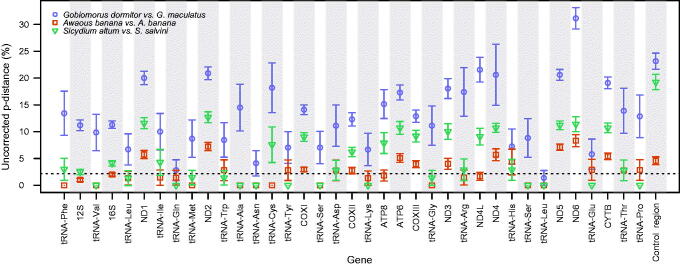
Genetic divergence across all mitochondrial genes between three pairs of species: *Gobiomorus dormitor* versus *G. maculatus*, *Awaous banana* from Atlantic versus *A. banana* from the Pacific watersheds, and *Sicydium salvini* versus *S. altum*. Genes are presented in the same order as they appear in these species mitochondrial genomes. Error bars represent standard errors and the dashed horizontal line indicates the commonly used threshold at 2% genetic divergence.

As would be expected, given they were not recovered as sister groups, *G. dormitor* and *G. maculatus* exhibited the largest divergences for every mitochondrial gene among the species compared (Figure 2). Distances were as low as 1.37% for tRNA Leu, and as large as 31.14% for ND6. On average, protein-coding genes were 18.74% ± 4.70 divergent. These genetic distances were also greater than between *G. dormitor* and *H. latifasciata* (mean = 15.43% ± 1.82), except for the COXII gene (12.74% versus 12.30%).

Throughout all comparisons, control region and ND6 were two of the most variable genes, while tRNA genes were consistently the least variable. The fewest differences existed between Atlantic and Pacific *A. banana*: ten tRNA genes were identical, with the remaining 12 ranging from 1.33 to 4.34% different. All protein-coding genes were over 2% divergent (mean = 4.75% ± 2.02), which is a common threshold for species delimitation in mitochondrial and barcoding studies (Ward 2009; Pereira et al. 2013; Bagley et al. 2015), except for ATP8 and ND4L (1.82% and 1.68% divergent, respectively). *Sicydium altum* and *S. salvini* divergences ranged between 1.66 and 12.70% for the protein coding genes (mean = 10.03% ± 1.66) (Figure 2).

The divergence values between *Awaous* and *Sicydium* geminate pairs are in the lower and upper boundaries of other geminate species pairs of peripheral fishes (e.g. *D. maculatus* versus *D. latifrons*: CYTB uncorrected p-distance = 8.6%, Galván-Quesada et al. 2016; and Caribbean *Agonostomus monticola* versus Pacific *A. monticola*: CYTB uncorrected p-distance = 8.3%, McMahan et al. 2013). Assuming that mutation rates are similar across evolutionary close species, these differences might reflect a range of divergence times across geminate species of peripheral fishes that could be related to their habitat preferences. For example, *Sicydium* species inhabit fast flowing rivers and creeks between sea level and 1100 m, whereas *A. banana* is commonly found in estuaries and rivers only up to 300 m above sea level. Therefore, the affinity of *Awaous* to near-shore environments and brackish waters may have allowed them to maintain gene flow until the very last instances of marine connectivity across the Central American Seaway. Overall, these new mitogenomes constitute an important resource for future investigations on rates of molecular evolution and divergence times of geminate species, and eventually for a further understanding of the geological history and age of the Isthmus of Panama.
